# Loss of Proliferation and Antigen Presentation Activity following Internalization of Polydispersed Carbon Nanotubes by Primary Lung Epithelial Cells

**DOI:** 10.1371/journal.pone.0031890

**Published:** 2012-02-27

**Authors:** Mandavi Kumari, Sumedha Sachar, Rajiv K. Saxena

**Affiliations:** 1 School of Life Sciences, Jawaharlal Nehru University, New Delhi India; 2 Faculty of Life Sciences and Biotechnology, South Asian University, New Delhi, India; University of Patras, Greece

## Abstract

Interactions between poly-dispersed acid functionalized single walled carbon nanotubes (AF-SWCNTs) and primary lung epithelial (PLE) cells were studied. Peritoneal macrophages (PMs, known phagocytic cells) were used as positive controls in this study. Recovery of live cells from cultures of PLE cells and PMs was significantly reduced in the presence of AF-SWCNTs, in a time and dose dependent manner. Both PLE cells as well as PMs could take up fluorescence tagged AF-SWCNTs in a time dependent manner and this uptake was significantly blocked by cytochalasin D, an agent that blocks the activity of acto-myosin fibers and therefore the phagocytic activity of cells. Confocal microscopic studies confirmed that AF-SWCNTs were internalized by both PLE cells and PMs. Intra-trachially instilled AF-SWCNTs could also be taken up by lung epithelial cells as well as alveolar macrophages. Freshly isolated PLE cells had significant cell division activity and cell cycling studies indicated that treatment with AF-SWCNTs resulted in a marked reduction in S-phase of the cell cycle. In a previously standardized system to study BCG antigen presentation by PLE cells and PMs to sensitized T helper cells, AF-SWCNTs could significantly lower the antigen presentation ability of both cell types. These results show that mouse primary lung epithelial cells can efficiently internalize AF-SWCNTs and the uptake of nanotubes interfered with biological functions of PLE cells including their ability to present BCG antigens to sensitized T helper cells.

## Introduction

Due to their unique structure and remarkable electrical and mechanical properties, carbon nanotubes (CNTs) have been used in wide applications in electronics, sensing, gas storage, aerospace, field-emission devices, catalytic supports, biomedical engineering and medical chemistry [1], [2], [3]. Structurally SWCNTs resemble rolled up tubes of graphite sheet of Sp2 hybridized carbon atoms, having a diameter of about 1 nm [4]. The nano dimension of SWCNTs with combination of high aspect ratio make them suitable candidates for new class of transporting vehicles for high cargo loading [5]. The increasing potential applications of SWCNTs raise the issue of potential toxic effects of these materials, as they are easily inhaled and become airborne due to low density and small size [6].

Several studies on the toxicity of carbon nanotubes have been reported in literature. A variety of SWCNT samples were tested with varying amounts of metal impurities and all SWCNT preparations were found to induce dose-dependent lung granulomas in mice [7]. A mild and transient pulmonary inflammatory response in rats instilled intra-tracheally with SWCNTs, with subsequent development of multifocal granulomas in the lungs was also reported [8]. The intratracheal instillation of pure SWCNTs resulted in granulomas, lung fibrosis and a significant elevation in markers of toxicity in broncho-alveolar lavage (BAL) fluid [9], and hence the authors concluded that SWCNTs exerted greater toxicity on a mass basis than crystalline silica. Immunosuppressive effect of SWCNTs resulting functional respiratory deficiencies and decreased bacterial clearance was also reported [10]. The acid functionalized SWCNTs (AF-SWCNTs) that are de-bundled and disperse easily in aqueous solution, were found to be highly toxic to the mouse lung epithelial cell line LA4 and induced strong pulmonary inflammation in mice [11]. AF-SWCNTs instilled intratracheally have also been shown to produce acute toxic effects in heart, suggesting that the nanotubes could traverse lung and reach heart [12].

While carbon nanotubes have toxic effects on lung epithelial cell lines, primary lung epithelial cells have not been examined for such effects. Recently, we have reported that mouse type I primary lung epithelial (PLE) cells have the ability to process and present mycobacterial antigens to sensitized T helper cells [13]. For these studies, we standardized the procedure for isolating and culturing mouse PLE cells. This has afforded us an opportunity to examine the toxic effects of carbon nanotubes on PLE cells. In the present communication, we show that acid functionalized poly-dispersed single-walled carbon nanotubes are taken up in a time and dose dependent manner by PLE cells and peritoneal macrophages. Cytochalasin D, an agent that blocks the acto-myosin dependent phagocytic activity of cells, partially blocked the uptake of AF-SWCNTs by PLE cells. Confocal microscopic studies confirmed that AF-SWCNT were internalized by PLE cells as well as PMs. PLE cells display proliferative activity in first few days of culture, which was blocked by exposure to AF-SWCNTs. Antigen presentation activity of PLE cells was also significantly inhibited by AF-SWCNTs. Taken together our results indicate that AF-SWCNTs are internalized by PLE cells, are toxic to them and interfere with their biological functions.

## Materials and Methods

### Animals

Inbred C57BL/6 mice (8–12 weeks old) were used throughout this study. Animals were bred and maintained in the animal house facility at Jawaharlal Nehru University, New Delhi or obtained from the National Institute of Nutrition, Hyderabad. The animals were housed in positive-pressure air conditioned units (25°C, 50% relative humidity). The JNU Institutional Animal Ethical Committee approved all experimental Protocols requiring the use of animals (IAEC Project Code: 5/2010).

### Reagents and other supplies

All tissue culture reagents, Collagenase, L-lysine, DNase, Trypsin, Tween-20, Tween 80 and Catalase were purchased from Sigma Aldrich (India). Sources of other reagents were: Fetal calf serum (FCS), Hyclone Laboratories Inc, USA; Middlebrook 7H11 Agar from Difco Laboratories, MI, USA. Dispase II, anti mouse CD16/32, anti mouse CD3 FITC, anti mouse CD45R FITC, anti mouse CD11b FITC, anti rat IgG2aκ FITC, anti rat IgG2bκ FITC, were purchased from BD Biosciences San Diego, CA, USA. Anti mouse F4/80 APC, rat serum and anti rat IgG2aκ APC were obtained from eBiosciences San Diego, CA, USA. Alexa Fluor 488 sodium hydrazide was purchased from Molecular Probes, USA. Mouse epithelial cell enrichment kit and mouse CD4 selection kit were obtained from Stem Cell Technology (USA). Costar (Cambridge, MA) was the source of all plastic disposable culture ware.

### Isolation and purification of primary lung epithelial (PLE) cells and peritoneal macrophages

Procedure used to isolate and culture lung epithelial cells has been described elsewhere [13]. Briefly, pieces of lung tissue from C57BL/6 mice were digested with dispase (2 mg/ml), collagenase (300 units/ml) and DNAse enzymes and the resulting cell suspension filtered through 40 µm cell strainer and washed. PLE cells were isolated by using the mouse epithelial cell enrichment kit containing immuno-magnetic beads and antibodies (anti-CD45, anti-TER119 and anti-CD31 to remove leukocytes, erythroid and endothelial cells respectively) by negative selection as recommended by the manufacturer (Stem Cell Technology, USA). PMs were also isolated from C57BL/6 mice. PMs and PLE cells were cultured in RPMI 1640 with 10% FBS containing 300 µg/ml Glutamine, 20 mM HEPES, 2×10^−5^ M 2-Mercaptoethyanol and 40 mg/ml Gentamycin. Cell cultures were incubated at 37°C with 5% CO_2_.

### Isolation and purification of CD4^+^ T cells

Spleen cells were derived from control or BCG immunized mice (1×10^6^ BCG, i.p. 22 days prior to sacrifice). CD4^+^ T cells were purified (above 99% purity) from the spleen cell suspensions by using mouse CD4 positive selection kit (Stem Cell Technology, USA) as described [13].

### Single-walled Carbon Nanotubes

Single-walled carbon nanotubes (SWCNTs) were procured from Sigma and were acid functionalized as described [11]. The particles (AF-SWCNTs) were dispersed in saline and sonicated for 2 min in Branson sonicator, before use. Detailed characterization of acid functionalized SWCNTs including elemental analysis, size and charge distribution, BET surface area and electron microscopic changes have been reported elsewhere [11], [12]. In summary, particle size distributions of AF-SWCNT aqueous suspensions obtained from the Zeta-sizer instrument showed that 95% of the particles were in the range between 22 and 138 nm. Mean zeta potential of AF-SWCNTs was – 57.2 mV and BET surface area was reduces by one third as compared to pristine SWCNTs. Basic tubular structure of AF-SWCNTs was intact though the sidewalls of nanotubes appeared roughened. In general, all changes were consistent with mild sulfonation/carboxylation of the nanotube sidewalls while leaving the basic nanotube structure unchanged.

### Attachment of fluorescent probes to AF-SWCNT

Fluorescent probe was covalently attached to the COOH groups on AF-SWCNTs as previously described [14]. Briefly, AF-SWCNTs were suspended in water and treated with 1-ethyl 3-(3-dimethyl aminopropyl) carbodiimide (EDAC) and N-hydroxysuccinimide (NHS) to get a succinimidyl intermediate. The mixture was continually shaken for 2 h and dialyzed extensively to remove excess NHS, EDAC and urea by-product. AF-SWCNTs thus activated were incubated with Alexa Fluor 488/633 hydrazide (Molecular Probes, Carlsbad, Ca) in dark with continuous mixing, followed by dialysis to remove free dye. Sephadex G25 column chromatography confirmed that the fluorescence tagged AF-SWCNT preparation, freshly prepared or after incubation in culture medium containing fetal calf serum for two days, were not contaminated with free probe (Results not shown).

### Confocal Microscopy

Internalization of AF-SWCNTs by PLE cells and PMs was confirmed by incubating PLE cells and PMs with alexa fluor 488 tagged AF-SWCNTs. PMs and PLE cells were grown on glass cover slips in tissue culture dishes in RPMI+10% Fetal Bovine Serum with fluorescenated AF-SWCNTs for 12 h and unbound/loosely bound particles removed by extensive washings with PBS. Cells were fixed with paraformaldehyde and analyzed on a Confocal Laser Scanning Microscope (Olympus FluoviewTM - FV1000 in AIRF, JNU).

### Antigen presentation assay

PLE cells and PMs were cultured (2×10^5^/well) in 96 well flat bottom plate. After overnight culture, cells were washed with complete medium to remove debris and dead cells and cultured in fresh culture medium for 2 days (peritoneal cells one day). Cells were treated with BCG sonicate (equivalent to a multiplicity of infection of 100∶1) for 24 h. After 24 h excess antigen was removed by washing and cells were fixed using 0.0125% glutaraldehyde for 30 sec followed by quenching with L-lysine [15]. Splenic CD4^+^ T cells (3×10^5^/well) cells from infected and uninfected mice were added to the wells containing fixed macrophages or epithelial cells. The amounts of IL-2 and IFNγ released in culture supernatants were assessed in cultured supernatant collected 24 and 48 h later respectively.

### Statistical analysis

Non-paired Students t-Test and ANOVA was used to test for significance of differences between different sets of data using Sigma plot software. Comparisons were considered significant at p≤0.05.

## Results

### Effect of AF-SWCNTs on cultured primary lung epithelial cells (PLE) cells and peritoneal macrophages (PMs)

**Figure 1:**
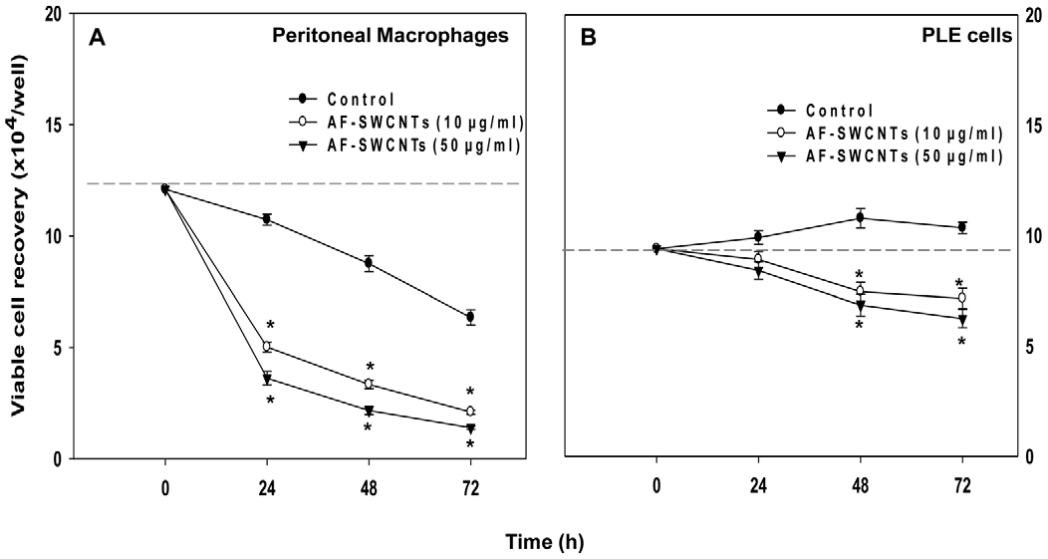
Effect of AF-SWCNTs on the recovery of PMs and PLE cells in culture. PMs and PLE cells were cultured in 48 well plate with or without AF-SWCNTs (10 or 50 µg/ml). After 24, 48 and 72 h, cells were washed, detached by trypsinization and suspended in 0.2% trypan blue solution in PBS. Recoveries of viable cell numbers of PMs (panel A) and PLE cells (panel B) were assessed by using a hemocytometer. Each point represents Mean ±SEM values obtained from 3 replicate assay wells. *p<0.05 for difference between control and AF-SWCNTs treated cell cultures.

Aim of the present study was to assess the biological effect of poly-dispersed AF-SWCNTs on PLE cells. PMs, with known phagocytic activity, were used in these experiments as positive controls. PLE cells and PMs were incubated with 10 or 50 µg/ml concentrations of AF-SWCNTs for up to 72 h and the recovery of live cells was assessed at different time points. There was a significant decline in recovery of PMs in the presence of all test doses of AF-SWCNTs (Figure 1A). In presence of 50 µg/ml of AF-SWCNTs, recovery of peritoneal macrophages decreased by approximately 66% at 24 h and 78% by 72 h. A significant decline in baseline cell recovery was noted in control cultures of PMs also. Results in Figure 1B showed that there was a marginal yet significant (p<0.05) increase in cell recovery from control cultures of PLE cells. AF-SWCNTs inhibited the recovery of PLE cells at both test doses (10, 50 µg/ml). Recovery of cells exposed to AF-SWCNTs (50 µg/ml) fell approximately by 15% as compared to the cell recovery in control cultures at 24 h, and further by 40% at 72 hour time point. For both PMs and PLE cells, toxic effect of 50 µg/ml dose was marginally more than the effect of lower 10 µg/ml dose (p<0.05) These results indicate that AF-SWCNTs preparations are toxic to both PLE cells as well as PMs in a time and dose dependent manner, though the effect appears to be more pronounced for PMs.

### Uptake of fluorescent tagged AF-SWCNTs particles by peritoneal macrophages and PLE cells

**Figure 2:**
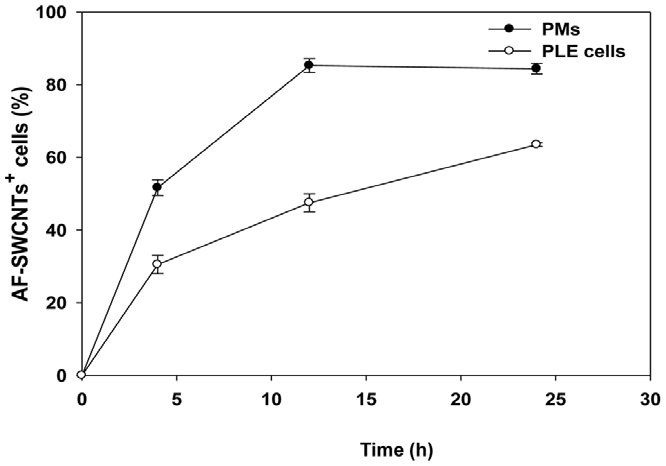
Uptake of fluorescence tagged AF-SWCNTs by PMs and PLE cells. Peritoneal macrophages and PLE cells were cultured in 48 well plates in presence of fluorescent tagged AF-SWCNTs (5 µg/ml). At different time periods, cells were washed with PBS and harvested by trypsinization. Percentage of cells positive for AF-SWCNTs was determined by using a flow cytometer. Each point represents Mean ±SEM of values obtained from 3 replicate assays.

**Figure 3:**
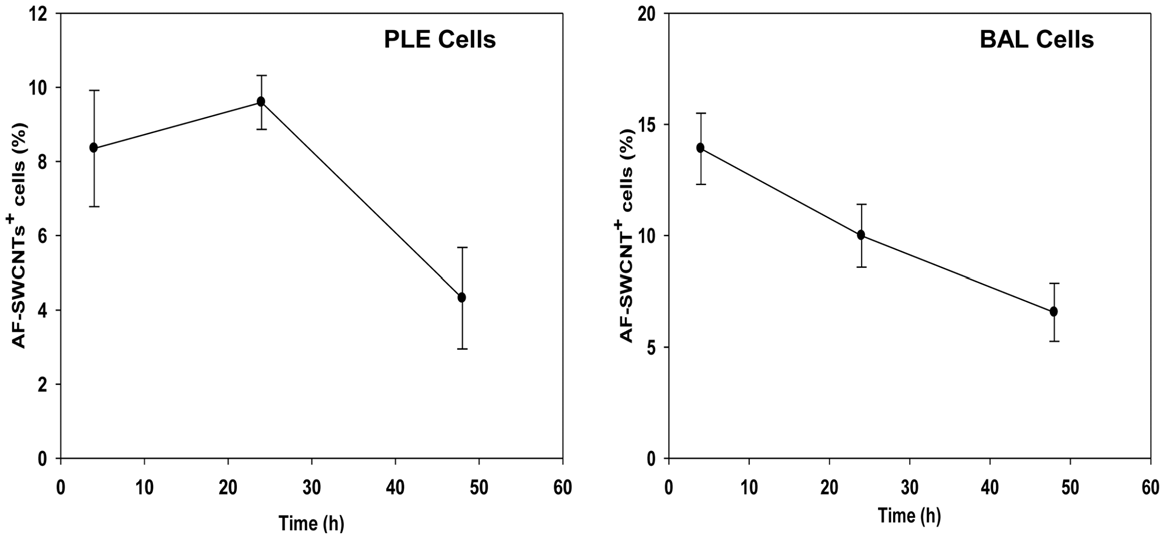
In vivo Uptake of fluorescence tagged AF-SWCNTs by lung epithelial cells and bronchoalveolar lavage (BAL) cells. Fluorescenated AF-SWCNTs (20 µg) were intratracheally administered in C57Bl6 mice. After 2, 24 and 48 h of the instillation of AF-SWCNTs, BAL cells were harvested and lung tissue processed for isolation of PLE cells. Uptake of AF-SWCNTs as percentage of cells positive for AF-SWCNT fluorescence was assessed flow cytometrically in PLE cells (left panel) and BAL cells (right panel). Each value represents Mean ± SEM of observations from 4 mice.

To study uptake of AF-SWCNTs by macrophages and PLE cells, AF-SWCNTs tagged with fluorescent probe, were added to PLE and PM cell cultures. At different time points, culture cells were harvested and examined by flow cytometry. Results in Figure 2 clearly indicate that with the progression of time, increased number of culture cells became associated with fluorescent AF-SWCNTs. At 4, 12 and 24 h time points, 30.63±2.51, 48±2.47 and 63±1.23% of PLE cells and 51.65±2.15, 85.24±1.88 and 84.37±1.48% of PMs respectively were found to be positive for AF-SWCNTs. These results indicate significant association of AF-SWCNTs with both PLE cells and PMs, though it was not clear at this stage if the association was due to the adherence of AF-SWCNTs with cells or by internalization of these particles. Uptake of AF-SWCNTs by lung epithelial cells as well as alveolar macrophages was also examined in vivo. Results in Figure 3 (panel A and B) show that 8% of the PLE cells and 14% of BAL cells were positive for AF-SWCNTs, 2 h after their intra-tracheal instillation in lungs. Percentage of AF-SWCNT^+^ cells declined at a later 48 h time point.

**Figure 4:**
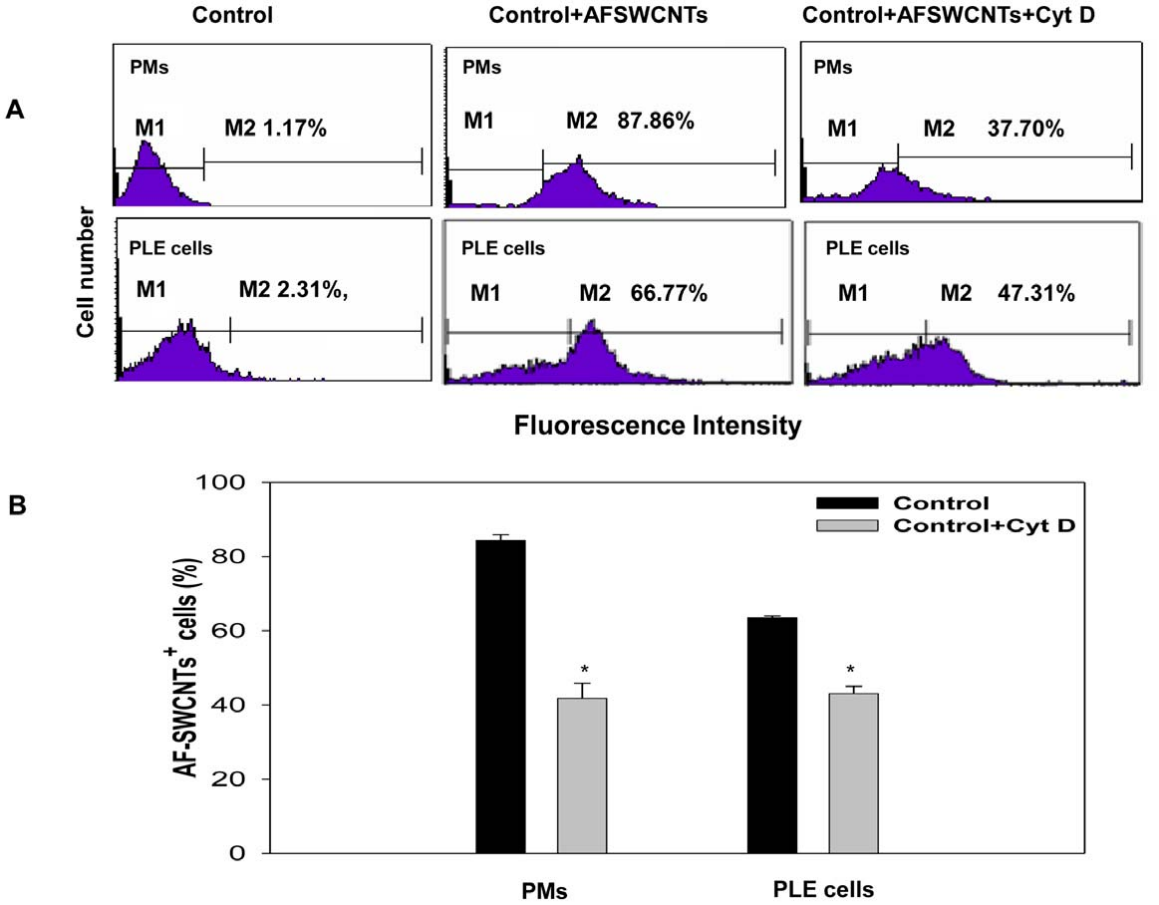
Effect of cytochalasin D on the uptake of AF-SWCNTs by PMs and PLE cells. PMs and PLE cells were cultured in 48 well culture plates in the presence or absence of 2.5 µg/ml cytochalasin D for 1 h. Fluorescence probe tagged AF-SWCNTs 5 µg/ml) were added and incubation continued for an additional 24 h. At the end of the incubation period, cells were washed with PBS, harvested by trypsinization and analyzed on FACS. Representative flow histograms for AF-SWCNTs uptake in control and cytolchalasin D treated PMs and PLE cells are shown in panel A where cells in M2 window represent AF-SWCNT^+^cells. Bar histograms in the lower panel represents Mean ± SEM of results obtained from 3 independent experiments.

To further understand the nature of association of AF-SWCNTs with PLE cells and PMs, the effect of cytochalasin D, an agent that blocks phagocytic activity, was tested on the uptake of AF-SWCNTs. Results in Figure 4 shows that as compared to control cells, AF-SWCNTs uptake by cytochalasin D treated macrophages and PLE cells was significantly lower. While 88% and 67% of control PMs and PLE cells were positive of AF-SWCNTs, the percentage fell to 38% and 47% respectively for cytochalasin D treated cells (Figure 4A). Mean uptake data in Figure 4B clearly shows a significant decline in AF-SWCNT uptake in cytochalasin D treated PLE cells and PMs. These results suggest that the uptake of AF-SWCNTs by both cells may at least partially be through an acto-myosin dependent mechanism responsible for phagocytic activity.

**Figure 5:**
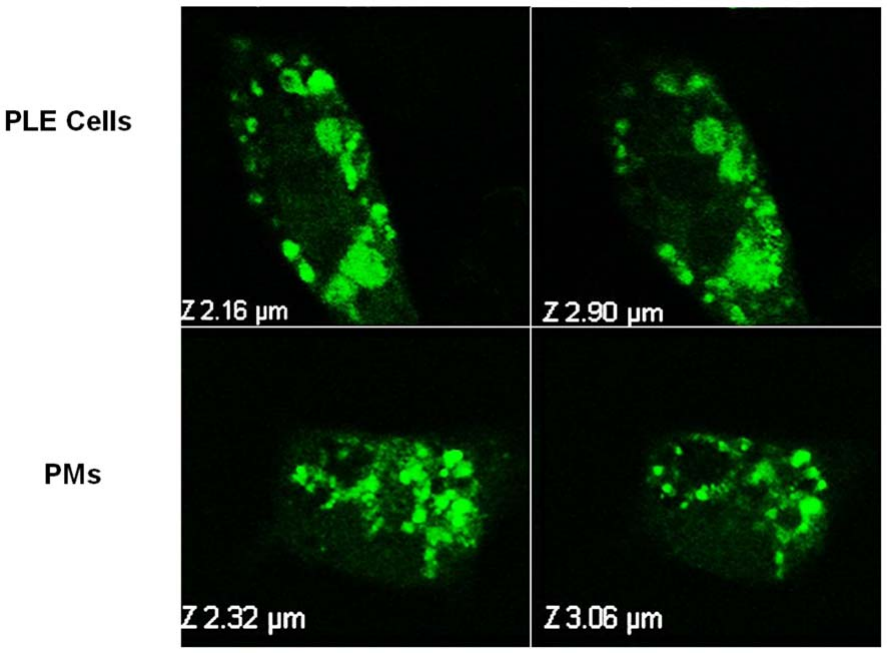
Examination by confocal microscopy of uptake of fluorescence tagged AF-SWCNTs by PMs and PLE cells. PMs and PLE cells were cultured on glass cover slip. After one day cells were washed with complete media and continued in culture for 2 more days. Fluorescence labeled AF-SWCNTs (5 µg/ml) were added to cell cultures and after 24 h cells were washed with PBS, fixed with paraformaldehyde, washed twice with quencher (Ammonium chloride) and examined by Confocal Laser Scanning Microscope. Two z-sections each of PLE cells (top two panels) and PMs (bottom two panels) show the presence of fluorescenated AF-SWCNTs in cytoplasm. (Magnification 60×).

Results of confocal microscopic examination (Figure 5) of PLE cells and PMs incubated with fluorescence tagged AF-SWCNTs clearly show the presence of fluorescence tagged AF-SWCNTs in the z-sections of both PLE cells and PMs, indicating internalization of AF-SWCNTs by both cell types.

### Effect of AF-SWCNTs on the cell cycle in primary lung epithelial cells

**Figure 6:**
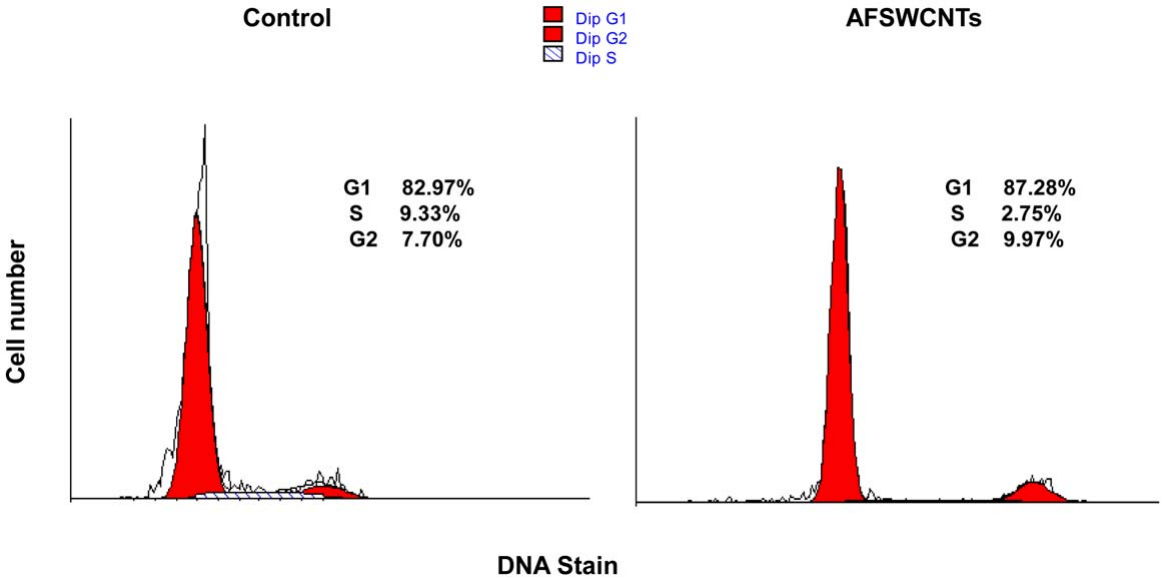
Effect of AF-SWCNTs on PLE cell cycle. Freshly isolated PLE cells were cultured in 6-well plate with or without of AF-SWCNTs (50 µg/ml). After 24 h, cells were washed and isolated by trypsinization. Cell were fixed, treated with RNase and stained with propidium iodide for flow cytometric analysis.. Data was analyzed by using Modfit software that enumerated proportion of cells in G1/Go phase (left peaks in all histograms), S phase (cross hatched peaks) and G2/M phase (right dark peaks in all histograms).

PMs are terminally differentiated macrophages that do not proliferate. PLE cells freshly isolated from mouse lungs however have a significant proliferative activity during first few days of culture. This has been confirmed by time-lapse micro videography (Video S1). Cell cycle analysis indicated that about 9.3% control PLE ells were in S-phase of cell cycle denoting proliferative activity (Figure 6). A marked decline of number of PLE cells in S-phase to 2.8% indicates that AF-SWCNTs exerted a marked anti-proliferative activity on PLE cells.

### Effect of AF-SWCNTs on BCG antigen presentation by PMs and PLE cells

**Figure 7:**
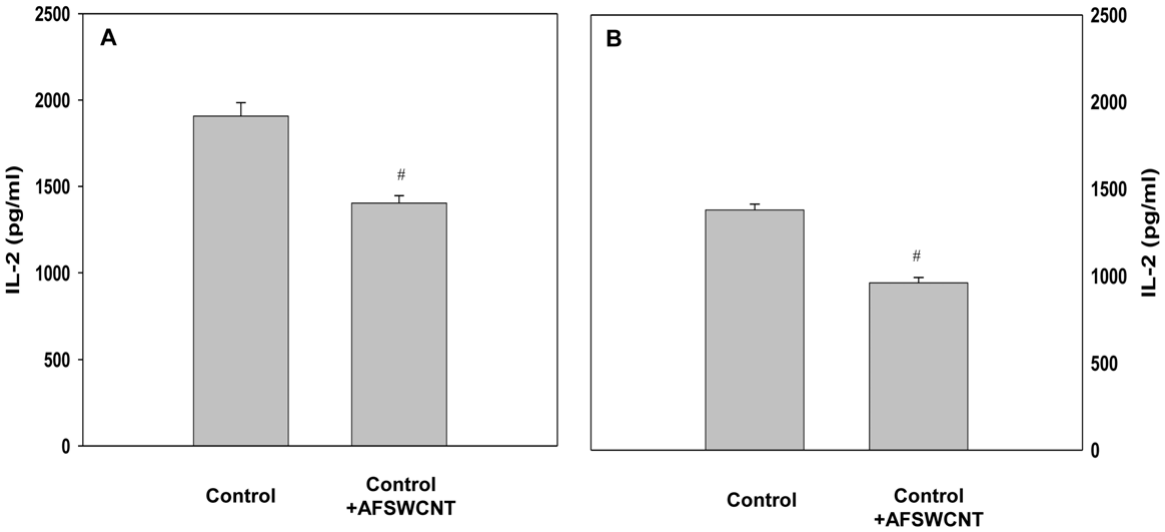
Effect of AF-SWCNTs on antigen presentation activity of PMs and PLE cells. Peritoneal macrophages (panel A) and PLE cells (panel B) (2×10^5^) were cultured with BCG sonicate (sBCG, equivalent to a MOI of 100∶1) with or without AF-SWCNTs (50 µg/ml) for 24 h. Excess antigen and particles were removed by washing and fixation was performed with glutaraldehyde and quenched with L-lysine. CD4^+^ T cells (3×10^5^ cells, purity >98%) isolated from spleens of BCG infected mice were added to the wells containing BCG pulsed PMs or PLE cells. The culture supernatants were collected after 24 h and the amount of IL-2 determined by ELISA. Each value represents Mean ± SEM of IL2 levels in 3 replicate assay wells. *(p<0.05).

We have previously shown that mouse PLE cells in culture are essentially type I epithelial cells and have the ability of processing and presenting BCG antigens to sensitized T helper cells [13]. Effect of treatment with AF-SWCNTs on antigen presenting ability of PLE cells and PMs was examined. For this purpose, PMs and PLE cells were cultured with BCG sonicate antigens for 1 day in presence or absence of AF-SWCNTs, washed and fixed with gluteraldehyde. Fixed PLE cells and PMs were incubated with purified BCG sensitized T-helper cells for 1 day and IL-2 secretion as an indicator of T cell activation was assessed in the culture supernatants. Results in Figure 7 (Panel A&B) show that AF-SWCNT treated PLE cells and PMs were significantly less efficient than control cells in inducing IL-2 secretion from T helper cells than control(p<0.05). These results suggest that AF-SWCNTs could lower the antigen presenting ability of both PLE cells and PMs.

## Discussion

Lung is the site of rapid exchange of oxygen and carbon dioxide gases that is crucial for sustaining life. It has the largest epithelial surface of the body that is constantly exposed to pathogens as well as environmental air containing air borne pollutants. Carbon nanotubes are potential air borne pollutants due to their respirable size and low density [6]. Several studies in literature have reported the adverse cellular and pulmonary reactions after pharyngeal or intratracheal instillation of SWCNT in rats or mice [4], [7], [8], [9], [11], [16], [17]. The aim of the present study was to investigate the interaction between AF-SWCNTs and primary lung epithelial (PLE) cells as well as macrophages.

We have previously shown that AF-SWCNTs have a marked toxic effect on a mouse lung epithelial cell line LA4. Incubation of LA4 cells with AF-SWCNTs resulted in a complete loss of cell proliferation activity and cell death [11]. In the present study, we have used primary lung epithelial (PLE) cells freshly isolated from mouse lungs, and found that a significant fall in the recovery of viable cells occurred when PLE cells were cultured with AF-SWCNTs. It is noteworthy that LA4 cells were markedly more sensitive to the toxic effects of AF-SWCNTs than the PLE cells, since AF-SWCNT preparation induced almost 100% cell death in LA4 cells [11] whereas at the same dose of AF-SWCNTs the recovery of viable PLE cells decreased only by 30% (Figure 1). Peritoneal macrophages (PMs) were more sensitive to AF-SWCNTs with a maximum decrease of about 90% in the recovery of viable cells. Thus, the toxic effect of AF-SWCNTs varies with the type of cells used.

Since unlike PLE cells, macrophages are professional phagocytes, greater toxic effect of AF-SWCNTs on PMs could be related to a greater degree of internalization of nanotubes. Uptake studies using fluorescence tagged AF-SWCNTs indeed indicated that PMs accumulated AF-SWCNTs at a relatively faster rate than the PLE cells, though the difference between the two cells tends to decline at later time points (Figure 2). Though PLE cells are not professional phagocytes, cytochalasin D treatment significantly reduced the uptake of AF-SWCNTs by PLE cells, indicating that the uptake of nanotubes was at least partially dependent on the acto-myosin musculature in PLE cell membrane; a feature of active phagocytosis. In a previous study, uptake of diesel exhaust particles by LA4 lung epithelial cell line as well as MHS alveolar macrophages was demonstrated and the uptake was blocked by cytochalasin D [18]. A partial blockade of uptake of AF-SWCNTs by cytochalasin D suggests that the uptake of AF-SWCNTs by PLE cells as well as PMs could occur through alternative mechanisms besides phagocytosis. Considering the shape of poly dispersed carbon nanotubes it is tempting to speculate that AF-SWCNTs could also enter the cells by piercing the cell membrane. In both PMs as well as PLE cells, internalization of AF-SWCNTs was confirmed by confocal microscopy since z-sections of cells incubated with fluorescence tagged AF-SWCNTs showed the presence of nanotubes in cellular cytoplasm. It should be noted that the cells as such are impermeable to free fluorescent probe (Life Technologies, http://products.invitrogen.com/ivgn/product/A30634) and for that reason, the presence of fluorescence in cellular cytoplasm could not be ascribed to the possibility that the probe detached from nanotubes entered the cells independently of the nanotubes. Accordingly, incubation of cells with free probe did not result in any staining of cells (Figure S1).

PLE cells freshly isolated from mouse lungs could proliferate in culture for first few days. This has clearly been shown by time lapse micro-videography (Video S1) and supported by the observation that the PLE cell numbers increased albeit marginally in culture conditions. Flow cytometric cell cycle analysis accordingly showed about 9% PLE cells in S-phase indicating that DNA replication took place in these cells. A marked decline in S-phase cells in PLE cells cultured with AF-SWCNTs indicate that the nanotubes internalized by PLE cells could interfere with the process of cell division. It may be speculated whether the loss of proliferative activity of PLE cells is related to the toxic effect of AF-SWCNTs on PLE cells. This however appears unlikely since unlike LA4 mouse epithelial cell line in which AF-SWCNTs induce marked apoptotic response and cell death [11], PLE cells were relatively resistant to similar toxic effects of AF-SWCNTs (Figure S2). Some decline noted in the recovery of PLE cells cultured in presence of AF-SWCNTs (Figure 1) may result from blockage of proliferative activity rather than due to induction of apoptosis and cell death.

We have recently shown that the mouse PLE cells have the ability to process and present mycobacterial antiges to antigen sensitized CD4^+^ T helper cells [13]. In the present study we further show that the antigen presenting ability of AF-SWCNT exposed PLE cells was significantly inhibited (Figure 7). Mechanism of inhibition of cell division as well as antigen presentation by AF-SWCNTs is not clear from our studies. In view of the fact that internalized AF-SWCNTs were localized in the cellular cytoplasm in a diffused manner, it is possible that that AF-SWCNTs inhibited cellular functions by interacting with multiple intracellular targets, though more work is needed to confirm this possibility.

## Supporting Information

Video S1 **Time lapse recording demonstrating the dividing ability of PLE cells.** PLE cells were isolated from C57Bl/6 mice and were cultured in RPMI+10%FBS at 37°C, 5% CO_2_ in tissue culture dish (35 mm). After 3 days in culture, PLE cells in culture dishes were placed on the observation platform of a live cell imaging (Nikon Eclipse Ti) microscope in a chamber maintained at 37 C in 5% CO2 atmosphere. Cell division activity was recorded for 12–14 h (WMV).
(WMV)

Figure S1 **Staining of MHS macrophage cell line with free fluorescence, Alexa fluor probe and fluorescence labeled AF-SWCNTs.** MHS-1 (mouse alveolar macrophage cell line) cells (0.2×10^6^/ml) were incubated with free Alexa fluor probe (0.2 µg/ml) or AF-SWCNTs coupled to the same probe (5 µg/ml) in RPMI+10%FBS for 8 h. Control and treated cells were washed with PBS, trypsinized, harvested and analyzed on flow cytometer. MHS cells treated with free probe (Blue histogram) and the control MHS cells (Black histogram) had only background fluorescence. Cells incubated with fluorescence probe tagged AF-SWCNT had significant stain (Pink).
(TIF)

Figure S2 **Lack of an apoptotic response in primary lung epithelial (PLE) cells to control and AF-SWCNTs.** PLE cells were cultured with or without AF-SWCNTs (50 µg/ml). After 24 h, cells were isolated by trypsinization, stained with 7AAD and Annexin V and analyzed by flow cytometry. Values in quadrangles in each histogram indicate the percentage of necrotic (7AAD^+^) and apoptotic (7AAD^−^ Annexin V^+^) cells in control and treated cell preparations.
(TIF)
