# Supplementary figures and images for: Loss of Proliferation and Antigen Presentation Activity following Internalization of Polydispersed Carbon Nanotubes by Primary Lung Epithelial Cells
Source: PLoS One. 2012 Feb 27;7(2):e31890. doi: 10.1371/journal.pone.0031890 (PMC3287983; doi:10.1371/journal.pone.0031890)

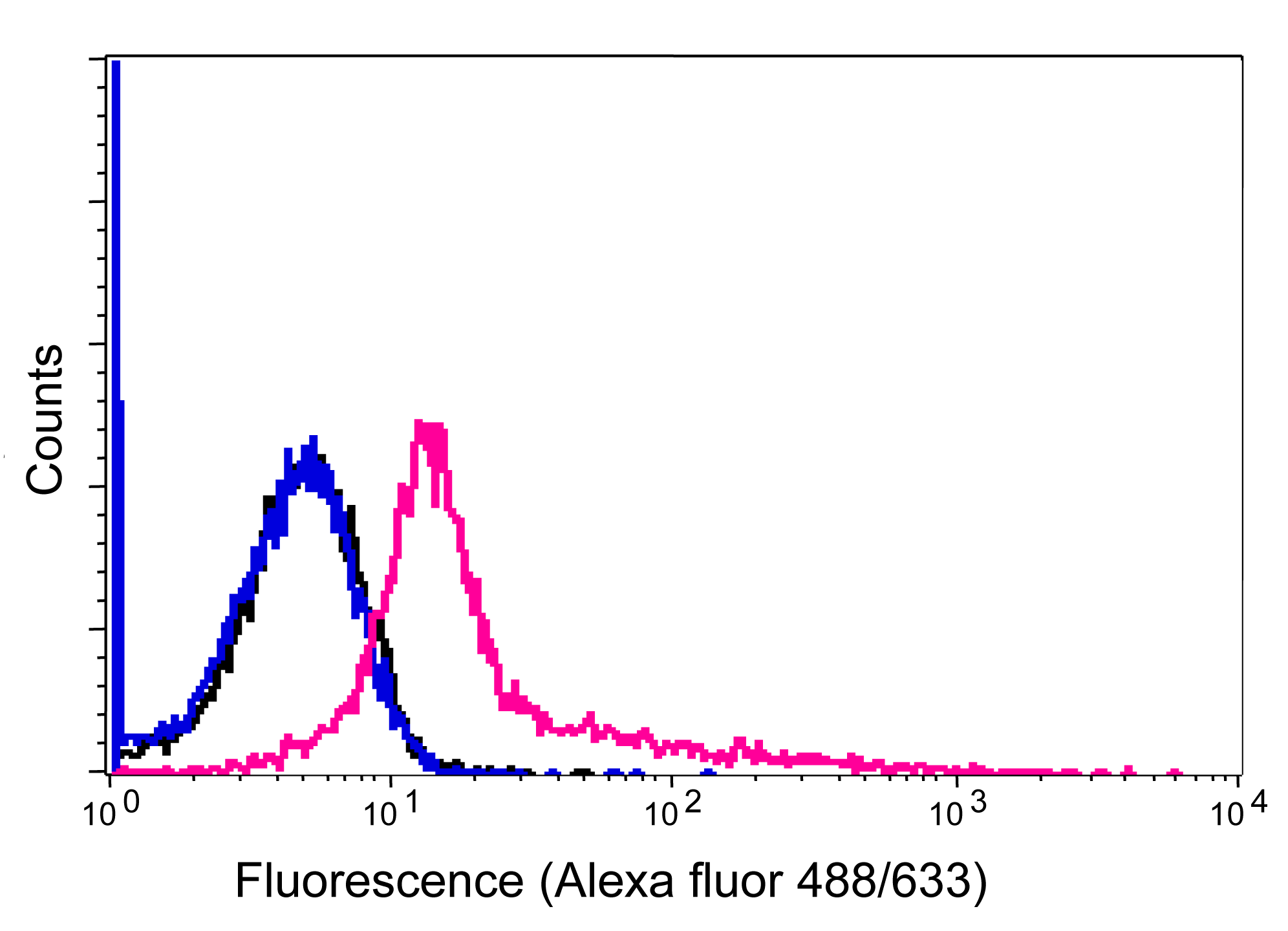

Supplement: Figure S1 — Staining of MHS macrophage cell line with free fluorescence, Alexa fluor probe and fluorescence labeled AF-SWCNTs. MHS-1 (mouse alveolar macrophage cell line) cells (0.2×10^6^/ml) were incubated with free Alexa fluor probe (0.2 µg/ml) or AF-SWCNTs coupled to the same probe (5 µg/ml) in RPMI+10%FBS for 8 h. Control and treated cells were washed with PBS, trypsinized, harvested and analyzed on flow cytometer. MHS cells treated with free probe (Blue histogram) and the control MHS cells (Black histogram) had only background fluorescence. Cells incubated with fluorescence probe tagged AF-SWCNT had significant stain (Pink). (TIF) [file pone.0031890.s002.tif]

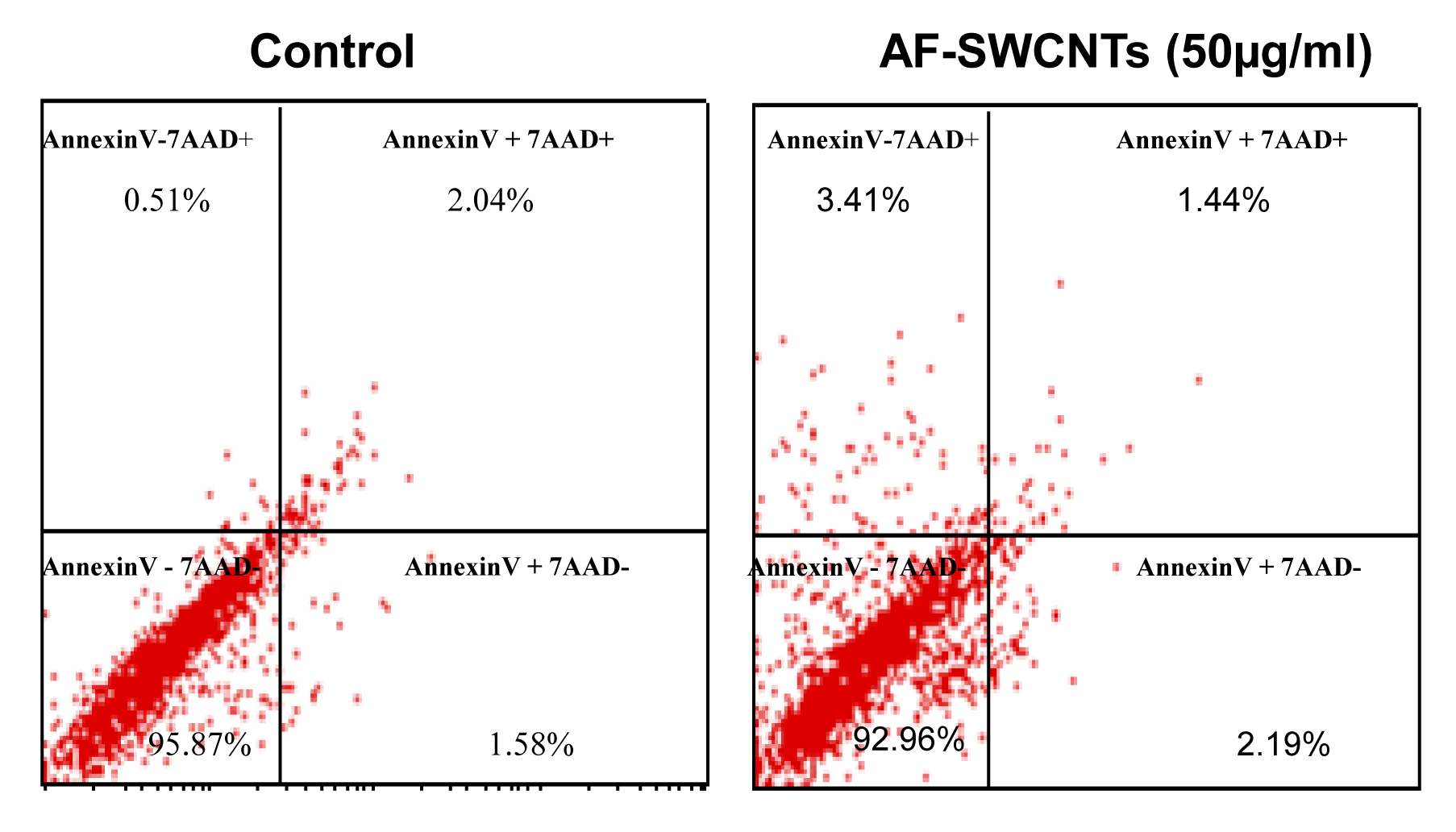

Supplement: Figure S2 — Lack of an apoptotic response in primary lung epithelial (PLE) cells to control and AF-SWCNTs. PLE cells were cultured with or without AF-SWCNTs (50 µg/ml). After 24 h, cells were isolated by trypsinization, stained with 7AAD and Annexin V and analyzed by flow cytometry. Values in quadrangles in each histogram indicate the percentage of necrotic (7AAD+) and apoptotic (7AAD− Annexin V+) cells in control and treated cell preparations. (TIF) [file pone.0031890.s003.tif]
